# Equitable access to a personalized treatment with the OncoCREAN national strategy in pediatric patients with acute lymphoblastic leukemia from the Mexican Social Security Institute

**DOI:** 10.3389/fonc.2024.1304640

**Published:** 2025-09-10

**Authors:** Enrique Lopez Aguilar, Angeles Del Campo Martinez, Ana Rioscovian Soto, Karen Ayala-Contreras, Marta Zapata-Tarrés, Miguel Enrique Cuéllar Mendoza, Yadira Betanzos Cabrera, Regina Zevada-Mosti, Sheyla Payán Romo, Oscar Omar Esquer Cota, Pamela Zazueta Mérida, Susana Elizabeth Anaya Aguirre, Emmanuel Rolando Rodríguez Cedeño, Maria De Lourdes Gutiérrez Rivera, Jorge Alfonso Martín Trejo, Elvia Yaroslab Mayorga Castillo, Carolina Contreras Camacho, Elio Aaron Reyes Espinoza, Norma Eryca Alatoma Medina, Hector Manuel Tiznado García, Christian Santillán Ávila, Jose Maria Sepúlveda Núñez, Sadya Fuerte Olvera, Lizeth Rodríguez Brindis, Jose Alvaro Parra Salazar, Lilia Adela García Stivalet, Lizette Bojórquez Steffani, Roberto Abraham Betancourt Ortiz, Aurora Treviño, Julio Mercado Castruita, Rocio Aburto Mejia, Adrian Jimenez Salinas, Francisco Javier Valdivia Ibarra, Fabiana Maribel Zepeda Arias, Ana Leticia Figueroa Rosas, Desiree Sagarnaga Durante, Fatima Borrego Perez, Jorge Humberto Urbina Ochoa, Gustavo Ignacio Prieto Torres, Jose De Jesus Chavez Martenez, Juan Gilberto Perez Soltero, Guillermo Careaga Reyna, Ma. Teresa Cristina Ramos Hernandez, Rocio Cardenas Navarrete, Elsa Fabiola Gonzalez Rosas, Adolfo Pineda Gordillo, Cecilia Ochoa-Drucker, Enrique López Facio, Mirna Guadalupe Rios Osuna, Claudia Selene Portillo Zavala, Sergio Ruben Cobo Ovando, A. B. Aguilar Román, Brenda Chávez Liñán, Benjamin Arroyo Acosta, Luis E. Bernabe-Gaspar, A. B. Rivera Ramírez, D. A. Malváez Estrada, E. Ulloa Salaices, V. P. Silva Delfín, G. González Villarreal, M. Estolando Ayón, Aaron Muñoz Flores, E. F. Ortiz de la O, H. J. Ortiz Trujillo, José Luis Moreno Domínguez, Miguel Lopez Suastegui, L. Gallegos Cruz, Celida Duque-Molina

**Affiliations:** ^1^ Coordinación de Atención Oncológica, Instituto Mexicano del Seguro Social, Ciudad de México, Mexico; ^2^ Coordinación de Investigación, Fundación IMSS, A.C, Ciudad de México, Mexico

**Keywords:** leukemia, Mexico, acute lymphoblastic leukemia, middle income countries (MIC), pediatric cancer

## Abstract

**Introduction:**

The Mexican Social Security Institute (IMSS) was created in 1943 as the main public institution in Mexico.

**Methods:**

In 2020, the OncoCREAN (Centros de Referencia Estatal para la Atención del Niño y de la Niña con Cáncer) strategy was established and consists of reference centers of Pediatric Oncology created to provide equitable access to pediatric patients with cancer in Mexico and toimprove the disease survival through standardized immunophenotyping, harmonized diagnosis, homogenized treatment, staff training, and complication response.

**Results:**

At 12 months of follow-up, overall survival is 89.9% in pediatric patients with acute lymphoblastic leukemia; we have no early death, and the main cause of death is still infection.

**Discussion:**

The current procedure in terms of immune molecular diagnosis is to send samples to the first national system of samples reference for the immunological diagnosis of leukemias based on immunophenotyping in the Cytomics Laboratory of the IMSS Research Center in Puebla, as a strong collaborative alliance. We present a narrative description of the strategy as preliminary results.

## 1 Introduction

The Mexican Social Security Institute (IMSS) was created in 1943 as the main public social institution with more than 80 million beneficiaries, including pediatric patients 18 years old or younger (1, 2). Forty-five percent of Mexican pediatric oncology patients are treated in IMSS, and the rest are treated in other social security institutions and the private system (3, 4). A national strategy was implemented through the IMSS National Oncologic Coordination to achieve equitable access and decreased mortality, regardless of the geographic area where patients live (1).

Pediatric cancer is a complex disease above all in the diagnostic and induction phases when oncological emergencies are more common and fatal. However, in the maintenance phase, patients are more stable, and treatment is generally less toxic. In countries like Chile, the first phase of treatment is given in the capital in a specialized hospital, and the maintenance phase is given in general hospitals closer to the patients’ home. Acute lymphoblastic leukemia (ALL) represents 48% of cancer diagnoses in childhood in Mexico. Overall survival is, on average, 57% in Mexico compared to 80%–90% in developed countries (5). For that reason, the strategy was implemented.

## 2 The OncoCREAN strategy

In the past, there were four main centers of clinical care for pediatric patients with cancer: two in Mexico City, one in Monterrey, and one in Guadalajara. The current organization involves the maintenance of these four centers with the addition of 32 areas inside pediatric hospitals where patients who already have their oncological diagnosis as well as the initial surgical treatment or induction therapy are treated. The implementation of the 32 centers was gradually carried out in approximately 24 months. Before 2020, the diagnosis of leukemia was done in each hospital when it was possible, or samples were sent to surrogate laboratories. Results in terms of survival toxicity and quality of life were local and not reported as an institutional study. Nowadays, all centers that receive patients with suspected leukemia are approached from the clinical aspect in the OncoCREAN. The bone marrow aspiration, as well as the staining, is done in the OncoCREAN to make the morphological diagnosis. In the same surgical time, the cytometry analysis sample is taken and sent to the West Biomedical Research Center (CIBIOR) located in the central area of Mexico. Samples arrive in less than 24 h via courier and are processed. The current procedure in terms of immune molecular diagnosis is to send samples to the first national system of samples reference for the immunological diagnosis of leukemias based on immunophenotyping in the Cytomics Laboratory of the IMSS Research Center in Puebla, as a strong collaborative alliance with the National Council of Humanities, Sciences, and Technologies (CONAHCYT). Thus, with the financial co-support from CONAHCYT and a new paradigm of research, the Cytomics Laboratory has been responsible for systematic sample preservation and exhaustive studies, for both diagnosis and measurable residual disease (MRD) as established international treatment protocols. Cytogenetics, according to the protocol, considers a DNA index of 1.16 or more and the translocation t (12,21) (ETV6-RUNX1) as provisionally classified as low-risk ALL. Patients with t (9,22) (BCR-ABL1) were considered to suffer from high-risk ALL, and the remaining patients were provisionally classified as having standard-risk (intermediate-risk) ALL.

The final risk status was determined based on the detection of MRD. Patients presenting with an MRD of 1% or more within the bone marrow aspirate on day 19 of remission induction or 0.10% to 0.99% after completion of 6 weeks of induction therapy were considered to have standard-risk ALL. MRD values of 1% or more after the completion of induction therapy indicated high-risk ALL. Immunophenotyping and classification of acute leukemia: BM samples were stained and acquired for flow cytometry analysis according to Euro Flow™ guidelines. First, samples were stained using the Acute Leukemia Orientation Tube (ALOT) to determine the lineage of immature blast cell populations. We classified AL into five review categories according to the affected cell lineage: Pro B-ALL (CD34+ CD19+ cyCD79a+), Pro B–Pre B-ALL (CD34−/+ CD19+ cyCD79a+), Pre B-ALL (CD34− CD19+ cyCD79a+), TALL (cyCD3+ smCD3lo CD7+), and AML (cyMPO+ or CD7+). Once the malignant hematopoietic lineage is identified, complementary antibody panels were applied (BCPALL, T-ALL, and AML). Sample acquisition was conducted using BD FACSCanto II or BD FACSLyric cytometers. Analysis of flow cytometry data was performed using Infinicyt 2.0 software.

When the diagnosis is made, each patient’s results are sent to the OncoCREAN in 24–72 h, the patient is stratified, and the induction therapy is planned and applied in one of the OncoCREAN centers. In January 2022, IMSS OncoCREAN started the implementation of the Total Therapy XV of St. Jude (TTXV) for leukemias that offers total access to and homogenization and decentralization of the treatment at a national level (6). The decision to choose the TTXV St Jude leukemia protocol was supported by the fact that Mexican pediatric oncologists, hematologists, and health professionals have strong experience due to the previous use of the Total XIII St Jude Leukemia protocol at a national level. All patients received the prednisone induction phase. Risk stratification was done as established in the TTXV protocol. Induction ended on day 33, while in this first-line treatment group, no patient received cranial radiotherapy. Toxicity was analyzed according to the National Cancer Institute classification.

The 36 centers have a pediatric oncologist/hematologist, a trained oncological nurse, and the resources to treat these diseases as well as their complications. All centers are connected to the National Coordination, having weekly communication to present new cases and reference stable patients. We are aware that, in the review, surveillance of all aspects of a patient’s life must be implemented, including how we evaluate the diagnosis, the timely treatment, and the presence of complications, nutrition, and psychological support. Pediatric oncologists/hematologists are the only ones responsible for the filing of documentations.

## 3 Results

So far, follow-up information is only 12 months. There were 809 patients, and the median age was 8.39 years old, with a minimum age of 4 months and a maximum age of 17 years. There were 471 male patients, with 468 being B-cell leukemias; 333 patients were classified as high-risk patients, 3 patients had central nervous system infiltration or mediastinal mass, and 6 patients relapsed. Overall survival is 89.9%; 82 patients died, the leading cause of which is infection. No deaths during the induction phase (early mortality) were reported.

There have been 72 online sessions with a frequency of one per week, in which all OncoCREANs have participated. Training in the protocol, the logistics of transporting samples, and the interpretation of diagnostic tests have been carried out. The current census indicates 91 oncologists and pediatric hematologists.

**Figure 1:**
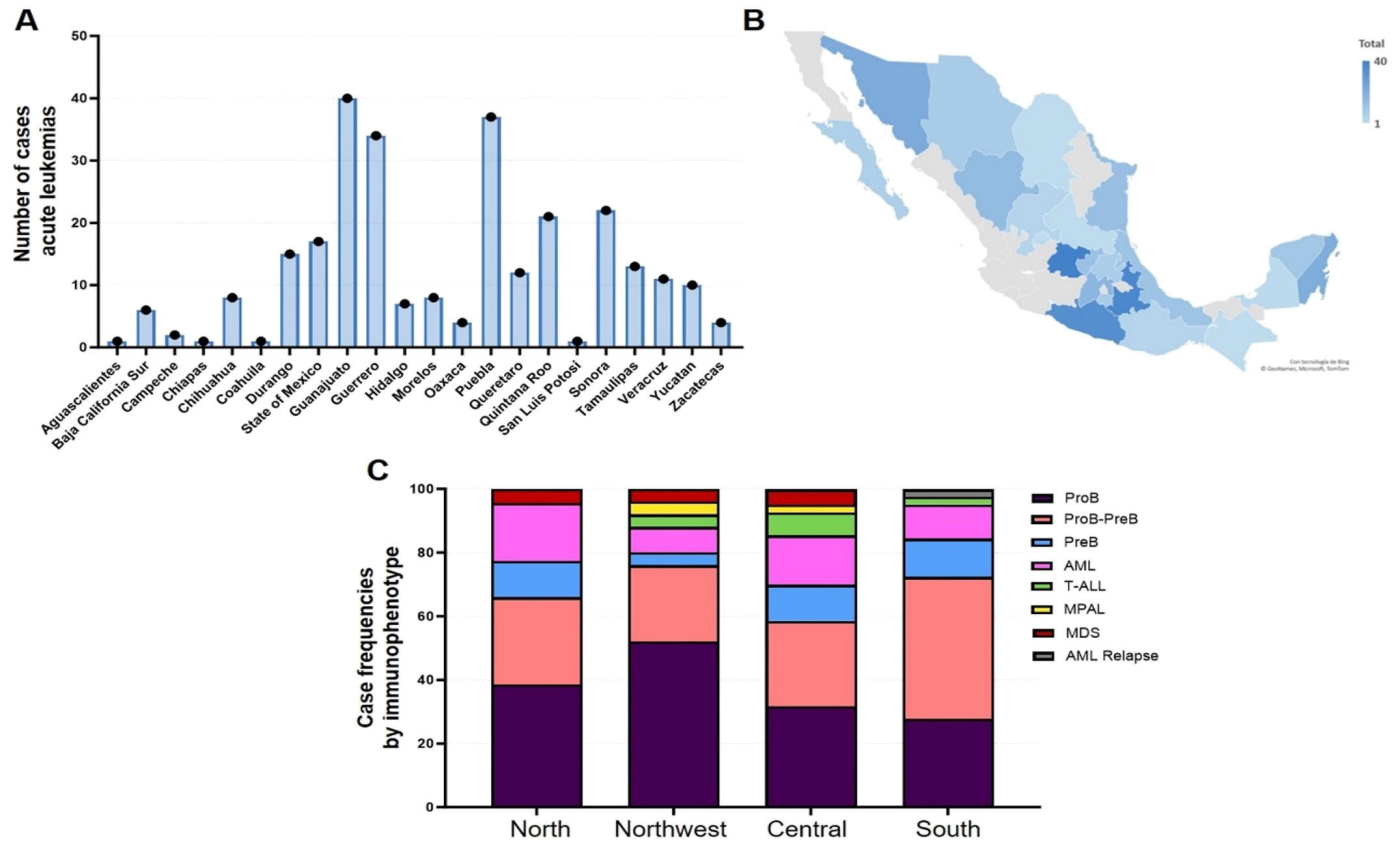
**(A, B)** represent distribution by states. **(C)** shows the leukemia by types and regions. AML, acute lymphoblastic leukemia; T-ALL, T-acute lymphoblastic leukemia; MPAL, mixed phenotype acute leukemia; MSD, myelodysplastic syndrome; AML relapse, acute lymphoblastic leukemia relapse.

From CIBIOR, 275 patients were studied. For logistics, the country is divided into four regions (North, Northwest, Central, and South). Most of our patients corresponded to Pro B or Pro B–Pre B cell leukemia as seen in **Figure 1**.

## 4 Discussion

In Mexico, this is a historic initiative because it brings patients with cancer equitable access to a standardized care protocol not only in the same care center but also at a national level in all IMSS hospitals. Considering that this has been described as a critical factor contributing to the increased survival of patients with ALL, we are sure that this strategy is changing the prognosis of this disease (7).

Death due to toxicity continues to be a determining factor, which is why in developing countries, such adaptations to chemotherapy protocols are made (7). It is imperative to intensify medical and nursing training of all OncoCREANs, emphasizing Golden Hour Pediatric Early Warning Score (PEWS) and the vascular line manual, which are strategies focused on early care of complications associated with treatment toxicity.

From 1950 to date, we have seen how systematization in treating patients with cancer has positively impacted survival. Despite this, we know that there are still global disparities in access to high-quality treatment for children with cancer (8). This work can be the beginning of a later analysis of how including patients with ALL in a standardized treatment protocol can allow us to make an individualized treatment according to the assigned risk and decrease the treatment’s long-term effects (6–10).

It would be the first time in Mexico that an institutional treatment strategy implemented at a national level is followed up with two main objectives: assistance and information generation for future research. In the IMSS, successful cooperation, central registration, precision, and quality of diagnosis and prognosis tests are changing the epidemiology of pediatric acute leukemias as well as the work organization. Analyzing the very preliminary information we have, early mortality is controlled probably due to the very close supervision of these patients in all the sections of health centers (emergency room, hospitalization, and outpatient clinic).

Considering the scalability of the strategy, we must consider the human infrastructure that at this moment at IMSS consists of 217 pediatric oncologists and 142 hematologists. On the other hand, cases of ALL were 800 per year with the number of cases increasing. The OncoCREAN system is designed to give attention to patients in the maintenance phase in which complications are less common to appear. This strategy has the advantage of patients being close to their homes; thus, the abandonment rate is decreasing (data not shown). Quality of life and school attendance must be studied in the future as social indicators of patient follow-up.

A formal survival analysis including all the variables associated with survival of patients with pediatric ALL was carried out and will be reported in a future study.

## 5 Conclusion

Implementing strategies to provide standardized diagnosis, prognosis, and treatment to patients with ALL are essential to improve survival (11). Moreover, training in support therapies for the care of complications secondary to treatment toxicity is indispensable. In review, OncoCREAN’s strategy in Mexico based on care and scientific research is bringing precision oncology closer to our pediatric population suffering from leukemia. This project has its share of challenges: this is one of the first attempts by IMSS to make a cooperative project at the national level at different centers, the time for the referral of samples at the CIBIOR, and the standardization of sample sending data. The strengths of this program include the fact that a national referral center for molecular diagnosis was made at the CIBIOR and the standardization of TTXV for the clinical centers.

## Data Availability

The raw data supporting the conclusions of this article will be made available by the authors, without undue reservation.
